# Regulatory mechanism of CCN2 production by serotonin (5-HT) via 5-HT_2A_ and 5-HT_2B_ receptors in chondrocytes

**DOI:** 10.1371/journal.pone.0188014

**Published:** 2017-11-16

**Authors:** Ayaka Hori, Takashi Nishida, Shogo Takashiba, Satoshi Kubota, Masaharu Takigawa

**Affiliations:** 1 Department of Biochemistry and Molecular Dentistry, Okayama University Graduate School of Medicine, Dentistry and Pharmaceutical Sciences, Okayama, Japan; 2 Department of Pathophysiology-Periodontal science, Okayama University Graduate School of Medicine, Dentistry and Pharmaceutical Sciences, Okayama, Japan; 3 Advanced Research Center for Oral and Craniofacial Sciences, Okayama University Dental School, Okayama, Japan; Kyungpook National University School of Medicine, REPUBLIC OF KOREA

## Abstract

Serotonin (5-hydroxytryptamine: 5-HT) is recognized as a neurotransmitter in the central nerve system and as a regulator of systemic blood pressure in the peripheral tissues. Recently, it was reported that 5-HT_2_ receptors (5-HT_2_Rs) were expressed in cartilage tissues lacking both vessels and neurons, suggesting possible novel functions of 5-HT during cartilage development and regeneration. Our previous data indicated that CCN family protein 2/connective tissue growth factor (CCN2/CTGF) plays a central role in cartilage development and regeneration. Therefore, the aim of this study was to investigate the effect of 5-HT on the production of CCN2 in chondrocytes. Firstly, we showed that the mRNAs of 5-HT_2_R subtypes 5-HT_2A_R and 5-HT_2B_R, were expressed in a human chondrocytic cell line, HCS-2/8; however, 5-HT_2C_R mRNA was not detected. In addition, exogenously added 5-HT did not affect the 5-HT_2A_R and 5-HT_2B_R expressions. Next, we demonstrated that CCN2 production was increased by treatment with a 5-HT_2A_R agonist and the combination of 5-HT and 5-HT_2B_R antagonist. In contrast, treatment with a 5-HT_2B_R agonist and the combination of 5-HT and 5-HT_2A_R antagonist decreased CCN2 production. Furthermore, we showed that phosphorylation of Akt and p38 MAPK were increased by treatment with 5-HT_2A_R agonist, and that phosphorylation of PKCε, PKCζ, ERK1/2 and JNK were increased by treatment with 5-HT_2B_R agonist. Finally, we found that 5-HT_2A_R was localized in the growth plate, whereas 5-HT_2B_R was localized in the articular cartilage. These findings suggest that 5-HT promotes CCN2 production through the 5-HT_2A_R in growth plates, and that it represses CCN2 production through the 5-HT_2B_R in articular cartilage for harmonized development of long bones.

## Introduction

Serotonin (5-hydroxytryptamine: 5-HT) is a monoamine produced from tryptophan, which is one of the essential amino acids [1, 2]. Two enzymes, tryptophan hydroxylase (TpH) and 5-hydroxytryptophan decarboxylase (5-HTpD), are involved in the biosynthetic pathway of 5-HT [1, 2]; and TpH is the rate-limiting enzyme in this pathway [1, 2]. TpH consists of 2 forms [1, 3], TpH1 and 2. It has been reported that 5-HT produced by TpH2, which is predominantly found in central nervous system (CNS), functions as a neurotransmitter in the CNS, and is also involved in the regulation of mood and appetite [1–3]. On the other hand, 5-HT produced by TpH1, which is mainly found in peripheral tissues, regulates numerous biological events including cardiovascular functions, bowel motility, vascular tone, and bladder control [1–3]. Most of the peripheral 5-HT is synthesized by the enterochromaffin (EC) cells in the bowel, taken up in platelets and is circulated throughout the body [1]. Eventually, peripheral 5-HT is released from platelets to serve as an endocrine factor when platelets become activated [2]. Because 5-HT does not pass the blood-brain barrier, it has been suggested that brain-derived 5-HT and peripheral tissue-derived 5-HT have distinct biological functions [1–3]. Previously, it was reported that platelet-derived 5-HT mediates liver regeneration [4] and that it directly inhibits osteoblast proliferation and bone formation [5]. These results suggest that peripheral tissue-derived 5-HT is involved in tissue regeneration.

It is a well-known fact that these multiple functions of 5-HT are exerted through 5-HT receptors on the cell membrane of many tissues [6]. Receptor families of 5-HT are divided into 7 subfamilies, which comprise as many as 15 subtypes identified up to now [2, 6]. Except for the 5-HT_3A_ and 5-HT_3B_ receptors, all other 5-HT receptors belong to the G-protein-coupled receptor superfamily, which is characterized by 7 membrane-spanning hydrophobic regions, N-terminal extracellular domains and C-terminal intracellular ones [6]. Although It has been believed that 5-HT receptors are expressed in the targeting tissues, such as neurons and vascular smooth muscle cells [6], it was reported that 5-HT_2_ receptor subtypes are expressed in cartilage anlage including the frontonasal mass and limb bud, which are not recognized as target tissues for 5-HT during early stages of embryonic morphogenesis [7, 8]. In addition, a recent study revealed that 5-HT is involved in autoimmune arthritis and bone resorption [9]. These findings suggest that 5-HT may play novel roles in cartilage development and regeneration, although cartilage tissues do not have nerve and vascular systems.

CCN protein 2/connective tissue growth factor (CCN2/CTGF) is a cysteine-rich heparin-binding protein with a molecular weight of 36–38 kDa [10–13]. This protein belongs to the CCN family, which consists of 6 distinct proteins [10–13]. CCN family proteins are involved in a number of biological processes in development, tissue repair, and tumor development and suppression [10–13]. Among them, CCN2 is a unique factor that is involved in cartilage development and regeneration [10–13]. Our early reports demonstrated that CCN2 is strongly expressed in the pre-hypertrophic region of the growth plate [14]. Subsequently, we also found that CCN2 is less expressed in articular cartilage tissues that are maintained without further growth [15]. Furthermore, using an *in vitro* culture system, we showed that recombinant CCN2 (rCCN2) enhances in the proliferation and differentiation of chondrocytes [16] and osteoblasts [17], as well as the differentiation of ostoclasts [18]. These findings suggest that CCN2 has multiple functions during cartilage and bone development. In particular, our *in vivo* study revealed that CCN2 promoted the regeneration of full-thickness defects, which defects from surface of articular cartilage reached bone marrow, in rat femoral cartilage [15]. On the basis of these findings, we hypothesized that 5-HT might regulate CCN2 production in chondrocytes, because both 5-HT and CCN2 are involved in the regeneration of cartilage tissues. To verify this hypothesis, we investigated whether 5-HT could directly regulate CCN2 production through the activation of 5-HT_2_ receptors in chondrocytes, principally by using agonists and antagonists of 5-HT_2_ receptors.

## Materials and methods

### Materials

Dulbecco’s modified Eagle’s medium (DMEM) and fetal bovine serum (FBS) were purchased from Nissui Pharmaceutical Co. Ltd. (Tokyo, Japan) and Nichirei Bioscience inc. (Tokyo, Japan), respectively. Plastic dishes and multi-well plates were obtained from Greiner Bio-One (Frickenhausen, Germany). Serotonin (5-hydroxytrptamin; 5-HT), BW723C86, which is an agonist of 5-HT_2B_R [19], and SB204741, which is an antagonist of 5-HT_2B_R [20], were purchased from Sigma (St. Louis, MO). Both ritanserin, which is an antagonist of 5-HT_2A/2C_R [21], and NBOH-2C-CN (4-[2-[[(2-hydroxyphenyl) methyl] amino] ethyl]-2,5-dimethoxybenzonitrile) hydrochloride, which is an agonist of 5-HT_2A_R [22], were obtained from Tocris Bioscience (Bristol, UK). Anti-5-HT_2A_R and anti-CCN2 antibodies were purchased from Abcam (Cambridge, UK), and anti-5-HT transporter (5-HTT) antibodies were from Alomone Labs (Jerusalem, Israel). Anti-extracellular signal-regulated kinase (ERK)1/2, anti-p38 MAPK, anti-c-Jun N-terminal kinase (JNK), anti-phospho-Akt, and anti-Akt antibodies were obtained from Cell Signaling Technology (Beverly, MA). Anti-phospho-ERK1/2, anti-phospho-p38 MAPK, and anti-phospho-JNK antibodies were from Promega (Madison, WI). Anti-5-HT_2B_R, anti-phospho-PKCα, anti-phospho-PKCε, anti-phospho-PKCζ, anti-PKCα, anti-PKCε, and anti-PKCζ antibodies were purchased from Santa Cruz Biotechnology (Santa Cruz, CA). Anti-β-actin and anti-Histone H3 antibodies were from Sigma and Epitomics Inc. (Burlingame, CA), respectively. Fluo 4-AM from Dojindo Laboratories (Kumamoto, Japan) was also employed.

### Cell cultures

Cells of the human chondrosarcoma-derived chondrocytic cell line HCS-2/8 [23, 24] were inoculated at a density of 4 x 10^4^ cells/cm^2^ into culture dishes containing DMEM supplemented with 10% FBS and cultured at 37°C in humidified air with 5% CO_2_.

### Real-time RT-PCR analysis

Total RNA was isolated from HCS-2/8 cells by using ISOGEN reagent (Nippon Gene, Tokyo, Japan). First-strand cDNA was synthesized with a primerScript^TM^ reverse transcriptase (RT) reagent kit (Takara Shuzo, Tokyo, Japan), and amplification reactions were performed under the following conditions: 95°C (5 s)-60°C or 65°C (10 s)-72°C (15 s) for 55 cycles by using a SYBR^®^ Green Real-time PCR Master Mix (Toyobo, Tokyo, Japan) and StepOne plus real-time PCR system (Applied Biosystems, Carlsbad, CA) as described previously [13]. After performing real-time PCR analysis, PCR products were analyzed by agarose gel electrophoresis. The nucleotide sequences of the primers and expected sizes of the amplicons are shown in Table 1.

**Table 1:** Human forward (F) and reverse (R) primers used for real-time PCR.

Gene	Accession No.	Primer sequence	Expected size (bp)
*COL2a1*	XM_017018831.1	(F) 5’-CAACAACCAGATTGAGAGCA-3’	166
		(R) 5’-CCATGTTGCAGAAAACCTTC-3’	
*ACAN*	NM_013227.3	(F) 5’-GGAGCAGGAGTTTGTCAACA-3’	186
		(R) 5’-CTTCTCGTGCCAGATCATCA-3’	
*CCN2*	NM_001901.2	(F) 5’-GCAGGCTAGAGAAGCAGACC-3’	152
		(R) 5’-ATGTCTTCATGCTGGTGCAG-3’	
*5-HT*_*2A*_*R*	NM_001165947.2	(F) 5’-TGGATCGGTTATCTCTCTTC-3’	149
		(R) 5’-AGCCGGTATTGTGTTCACTA-3’	
*5-HT*_*2B*_*R*	NM_001320758.1	(F) 5’-GCACTGGGCAGCTCTTCTGA-3’	149
		(R) 5’-CCAACCAGCAAATCAGCCAC-3’	
*5-HT*_*2C*_*R*	NM_001256760.2	(F) 5’-TTTCAATTCGCGGACTAAGG-3’	96
		(R) 5’-GTCCCTCAGTCCAATCACAG-3’	
*TpH-1*	NM_004179.2	(F) 5’-TGCAAAGGAGAAGATGAGAGAATTTAC-3’	113
		(R) 5’-CTGGTTATGCTCTTGGTGTCTTTC-3’	
*GAPDH*	NM_001289746.1	(F) 5’-GCCAAAAGGGTCATCATCTC-3’	214
		(R) 5’-GTCTTCTGGGTGGCAGTGAT-3’	

### Western blot analysis

HCS-2/8 cells were treated with 5-HT, one of the agonists, or a combination of 5-HT and an antagonist. After 24 h, the cell lysates were prepared, and Western blot analysis was performed as described previously [13]. Briefly, proteins isolated from these cells were separated by sodium dodecyl sulfate polyacrylamide gel electrophoresis (SDS-PAGE) and were then transferred to polyvinylidene difluoride (PVDF) membranes (Millipore) by using a semi-dry transfer apparatus (Atto Corp., Tokyo, Japan). Blots were then reacted overnight at 4°C with primary antibodies used at a predetermined dilution. Then, after washing with Tris-buffered saline-Tween 20 (TBST) and TBS buffers, the blots were incubated for 60 min at room temperature with secondary antibodies conjugated with horseradish peroxidase (HRP). Subsequently, the membranes were washed with TBST and TBS buffers, and the bands were detected with the chemiluminescence substrate by using a LAS-4000 mini image analyzer (Fuji Film, Tokyo, Japan). The band intensities were determined by using Multi Gauge ver. 3.0 soft (Fuji Film).

### Detection and measurement of intracellular Ca^2+^

After HCS-2/8 cells had reached sub-confluence, they were pre-treated with Fluo 4-AM probe (Dojindo) in recording medium (20 mM HEPES, 115 mM NaCl, 5.4 mM KCl, 0.8 mM MgCl_2_, 1.8 mM CaCl_2_, 13.8 mM glucose, pH 7.4) for 20 min. The culture medium was then replaced with recording media without Fluo 4-AM, and the cells were treated with 5-HT or agonists for 1 min. Calcium influx was observed by fluorescence microscopy as described previously [25]. For measurement of intracellular Ca^2+^, HCS-2/8 cells were inoculated into 96 well black plate with clear bottom (BD Biosciences; Bedford, MA) and cultured for 3 days. Then, after HCS-2/8 cells were pre-treated with Fluo-4-AM probe, they were treated with 5-HT or agonists, and the probe was immediately excited at a wavelength of 485 nm. The fluorescence intensity at 535 nm was measured collected using a Fluoroskan Ascent FL (Thermo Labsystems; Helsinki, Finland).

### Enzyme-linked immunosorbent assay (ELISA) for 5-HT

HCS-2/8 cells were treated with 5-HT for 24 h. The cell lysate and conditioned medium were harvested, and 5-HT concentrations were determined using a commercial ELISA kit (Enzo Life Sciences; Farmingdale, NY), following the manufacturer’s instructions. Samples from 3 independent experiments were analyzed in duplicate, and the mean and standard deviation were calculated.

### Immunohistochemical analysis

Male C57BL/6 mice were housed in filter-top cages with paper-chip bedding under specific pathogen free conditions in an inverted 12 h light/dark cycle in a humidity-temperature controlled environment, and were provided with standard diet and water *ad libitum* [26]. The mice at 60-day-old were euthanized (S1 Checklist: NC3Rs ARRIVE Guidelines Checklist 2014). Then, whole knee joints were dissected and fixed in 10% formalin overnight at 4°C. Next, the tissues were decalcified with 0.5 M ethylendiamine tetraacetic acid (EDTA) for 3 weeks and subsequently embedded in paraffin. Frontal sections of 7-μm-thickness were mounted on silane-coated slides, deparaffinized, and treated with hyaluronidase (25 mg/ml) for 30 min at room temperature for epitope retrieval. Then, immunohistochemistry was performed with a Histofine kit (Nichirei; Tokyo, Japan), as described previously [13]. Color was developed with diaminobenzidine (DAB), and sections were counterstained with methyl green. The Animal Committee of Okayama University Graduate School of Medicine, Dentistry, and Pharmaceutical Sciences approved all of the procedures.

### Statistical analysis

Unless otherwise specified, all experiments were repeated at least twice and similar results were obtained. Normality testing (F-test) was performed for all experiments. After confirmation of normality, statistical analyses were performed by using Tukey’s test, Dunnett’s test or Bonferroni’s test to compare the means of multiple groups or by using an unpaired Student’s *t*-test to compare the means of 2 groups. All data were presented as the mean and standard deviation.

## Results

### Detection of 5-HT_2_ receptors and regulation by 5-HT in HCS-2/8 cells

**Fig 1:**
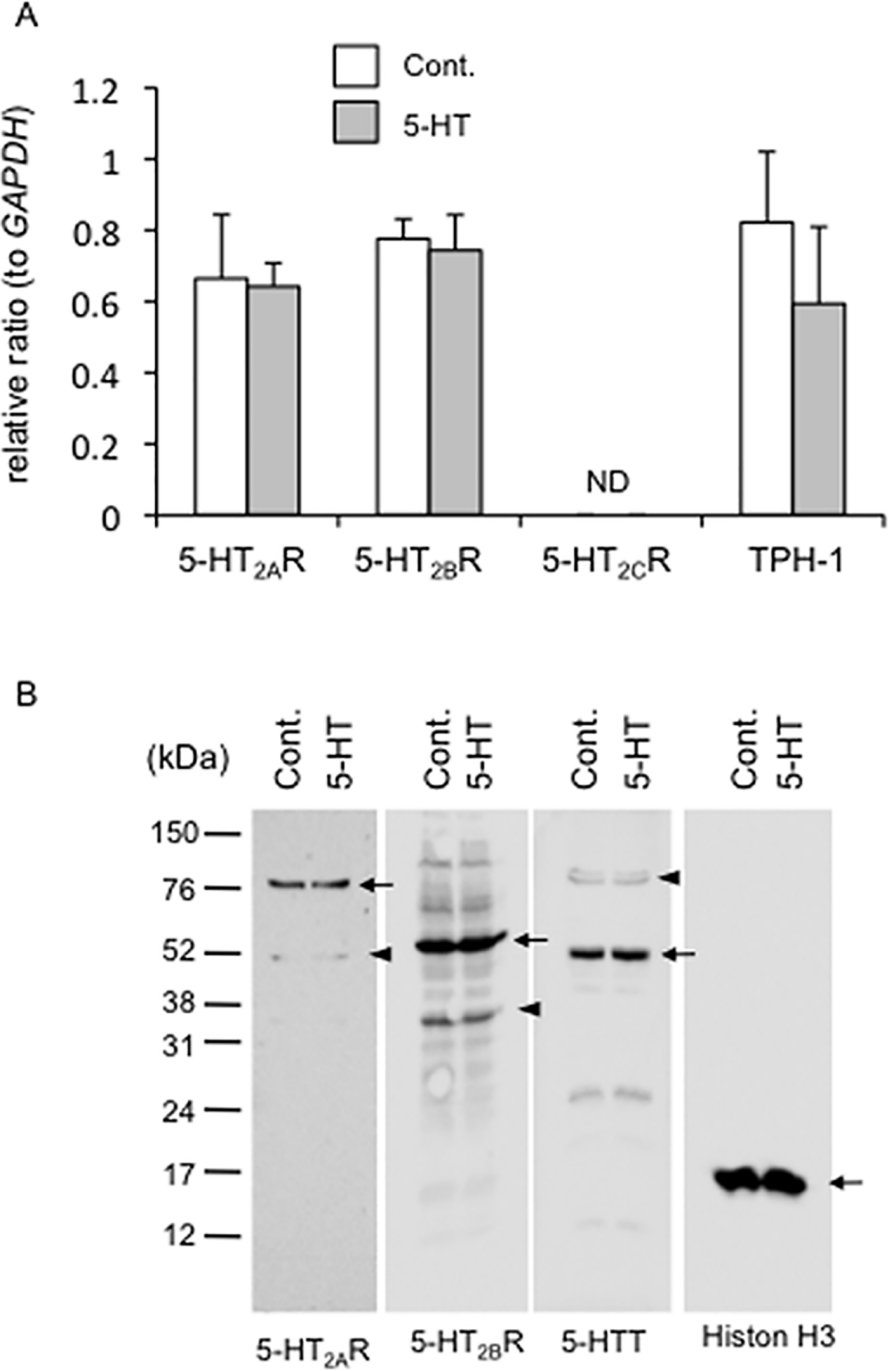
Effect of 5-HT on the gene expression and protein production of 5-HT_2_ receptor in HCS-2/8 cells. (A) After HCS-2/8 cells had reached confluence, they were treated with 5-HT at a concentration of 10 μM for 24 h. Then, total RNA was isolated and real-time RT-PCR analysis was performed by using specific primers for GAPDH, TpH-1, 5-HT_2A_R, 5-HT_2B_R and 5-HT_2C_R. The ordinate indicates the relative ratio with respect to GAPDH expression, and data represents mean and standard deviation of culture with (n = 4) or without 5-HT (n = 4). The gene expressions levels of *5-HT*_*2A*_*R*, *5-HT*_*2B*_*R*, and *TpH-1* were confirmed in HCS-2/8 cells, whereas 5-HT_2C_R was not detected (ND). In addition, these gene expressions were not affected by the treatment with 5-HT. (B) After HCS-2/8 cells had reached confluence, they were treated with 5-HT for 24 h. Then, Western blot analysis was performed by using antibodies recognizing the indicated proteins. The apparent molecular weights of 5-HT_2A_R were 75 kDa (arrow) and 50 kDa (arrowhead). The major band of 5-HT_2B_R indicated a molecular weight 55 kDa (arrow), and minor bands were found at 30 kDa (arrowheads). The major band of 5-HTT was at 53 kDa (arrow), and a minor band at 75 kDa (arrowhead). Exogenous 5-HT added had no effect on the production of these proteins. Histone H3 was used as a loading control.

To investigate whether the genes of 5-HT_2A_R, 5-HT_2B_R and 5-HT_2C_R were expressed in HCS-2/8 cells, we firstly performed real-time PCR analysis in HCS-2/8 cells treated with 5-HT at the concentration of 10 μM for 24 h. As shown in Fig 1A, in HCS-2/8 cells, gene expression of 5-HT_2A_R and 5-HT_2B_R was detected, whereas that of 5-HT_2C_R was not. In addition, mRNA of *TpH-1* was also expressed in the cells. Moreover, exogenously added 5-HT had no effect on the regulation of these gene expressions. Next, to confirm the production of 5-HT_2A_R, 5-HT_2B_R and 5-HTT proteins in HCS-2/8 cells treated with 5-HT, we performed Western blot analysis by using specific antibodies against them. It has been reported that the 5-HT_2A_R contains 5 potential N-linked glycosylation sites in its extracellular N terminus [27, 28] and that the receptor is expressed as a 75-kDa protein when N-glycosylated and as a 50-kDa one without N-glycosylation [28, 29]. In this study, the 75-kDa receptor was the prominent form of the 5-HT_2A_R in HCS-2/8 cells, although the 50-kDa form was also slightly detected. In addition, the most commonly reported molecular weight of the 5-HT_2B_R is 55 kDa [29], and we also detected the major immnoreactive band visualized by anti-5-HT_2B_R antibody at approximately ~55 (55) kDa in these HCS-2/8 cells (Fig 1B). Our immunoblotting data obtained with anti-5-HT_2B_R antibody revealed minor bands at approximately 30 kDa. We suspect that the band appears at 30 kDa may be a degradation product during sample preparation, or a nonspecific band. Furthermore, we found a major band at approximately 53 kDa and a minor one at approximately 75 kDa by Western blotting analysis when using anti-5-HTT antibody (Fig 1B). As the proteins of 90, 65, and 60 kDa (transporter with N-glycosylation) and the protein of 50 kDa (non-glycosylated transporter) are reported as being 5-HTT [30], our data suggest that the non-glycosylated form of the 5-HTT may be the major form in HCS-2/8 cells. Alternatively, it is possible that the antibody primarily which used in this study mainly recognizes the nonglycosylated protein. These results indicate that HCS-2/8 cells produce 5-HT_2A_R, 5-HT_2B_R and 5-HTT proteins, and that 5-HT added onto the cells has no effect on the production of these proteins (Fig 1B).

### Gene expression and protein production of CCN2 regulated by 5-HT via 5-HT_2A_R or 5-HT_2B_R in HCS-2/8 cells

**Fig 2:**
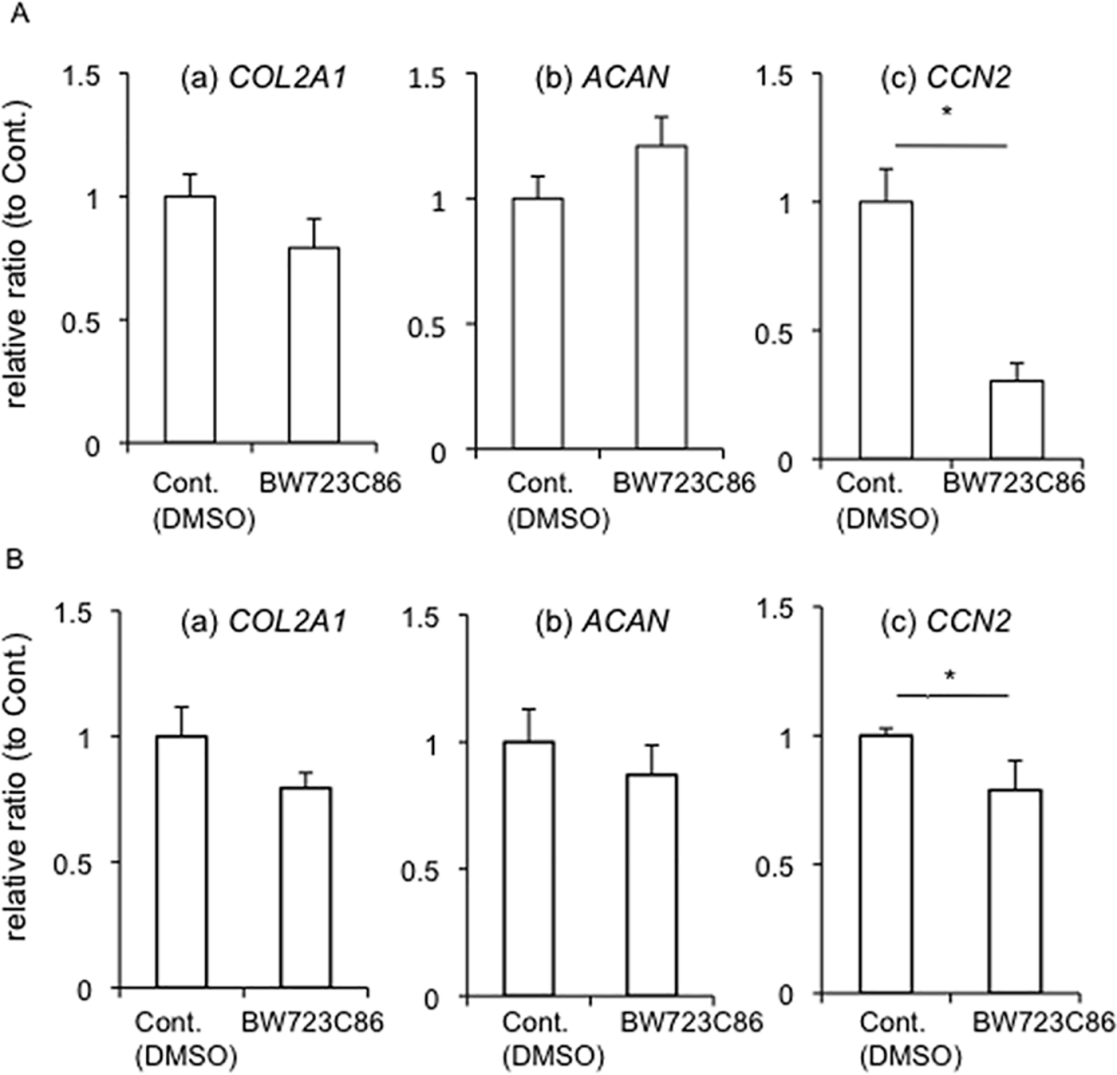
Real-time RT-PCR analysis of *COL2A1*, *ACAN*, and *CCN2* mRNAs in HCS-2/8 cells treated with BW723C86, an agonist of 5-HT_2B_R. (A) After HCS-2/8 cells had reached confluence, they were treated with BW723C86 at a concentration of 10 μM for 12 h. Then, total RNA was collected; and real-time RT-PCR analysis was performed. The amounts of these mRNAs were normalized to that amount of *GAPDH* mRNA. The graphs show the expression levels of (a) *COL2a1*, (b) *ACAN*, (c) *CCN2* after incubation with (n = 6) or without BW723C86 (n = 6). (B) HCS-2/8 cells were treated with BW723C86 at a concentration of 10 μM for 24 h. The graphs show the expression levels of (a) *COL2a1*, (b) *ACAN*, (c) *CCN2* after incubation with (n = 3) or without BW723C86 (n = 3). In all graphs, the ordinate indicates the relative ratio with respect to untreated sample (ratio = 1.0), and bars represent mean and standard deviation. The data were analyzed by Student *t*-test, and *p* < 0.05 (*) was considered significant.

**Fig 3:**
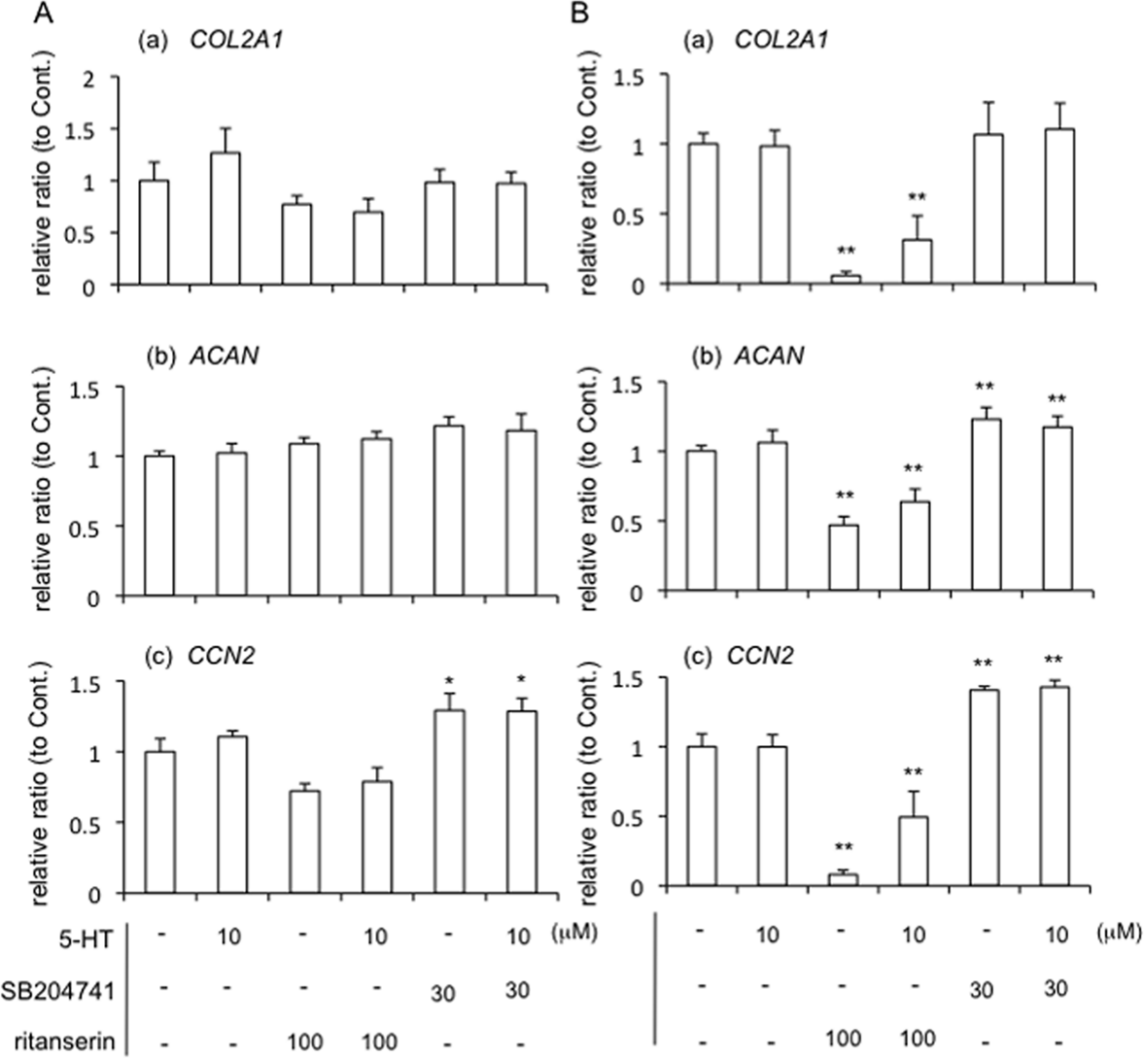
Real-time RT-PCR analysis of *COL2A1*, *ACAN*, and *CCN2* mRNAs in HCS-2/8 cells treated with the combination of 5-HT and SB204741, an antagonist of 5-HT_2B_R or retanserin, an antagonist of 5-HT_2A_R. (A) HCS-2/8 cells were grown until they had reached confluence. Then, the cells were treated with 5-HT (10 μM), ritanserin, an antagonist of 5-HT_2A_R (100 μM), SB204741, an antagonist of 5-HT_2B_R (30 μM) or the combination of 5-HT and SB206553 or ritanserin. After 12 h, total RNA was collected; and real-time RT-PCR analysis was then performed. The amounts of these mRNAs were normalized to that amount of *GAPDH* mRNA. The graphs show the expression levels of (a) *COL2a1*, (b) *ACAN*, (c) *CCN2* mRNAs in HCS-2/8 cells treated with vehicle control (PBS and/or DMSO, n = 4), 5-HT (n = 4), ritanserin alone (n = 3), the combination of 5-HT and ritanserin (n = 3), SB204741 alone (n = 4), or the combination of 5-HT and SB204741 (n = 4). (B) HCS-2/8 cells were treated with 5-HT, ritanserin, SB204741, or the combination of 5-HT and SB206553 or ritanserin for 24 h. The graphs show the expression levels of (a) *COL2a1*, (b) *ACAN*, (c) *CCN2* in HCS-2/8 cells treated with vehicle control (PBS and/or DMSO, n = 4), 5-HT (n = 4), ritanserin alone (n = 4), the combination of 5-HT and ritanserin (n = 4), SB204741 alone (n = 4), or the combination of 5-HT and SB204741 (n = 4). In all graphs, the ordinate indicates fold induction with respect to control sample (ratio = 1.0), and bars represent mean and standard deviation. The data were analyzed by Tukey’s test for multiple comparisons, and *p* < 0.05 (*), *p* < 0.01 (**) was considered significant.

**Fig 4:**
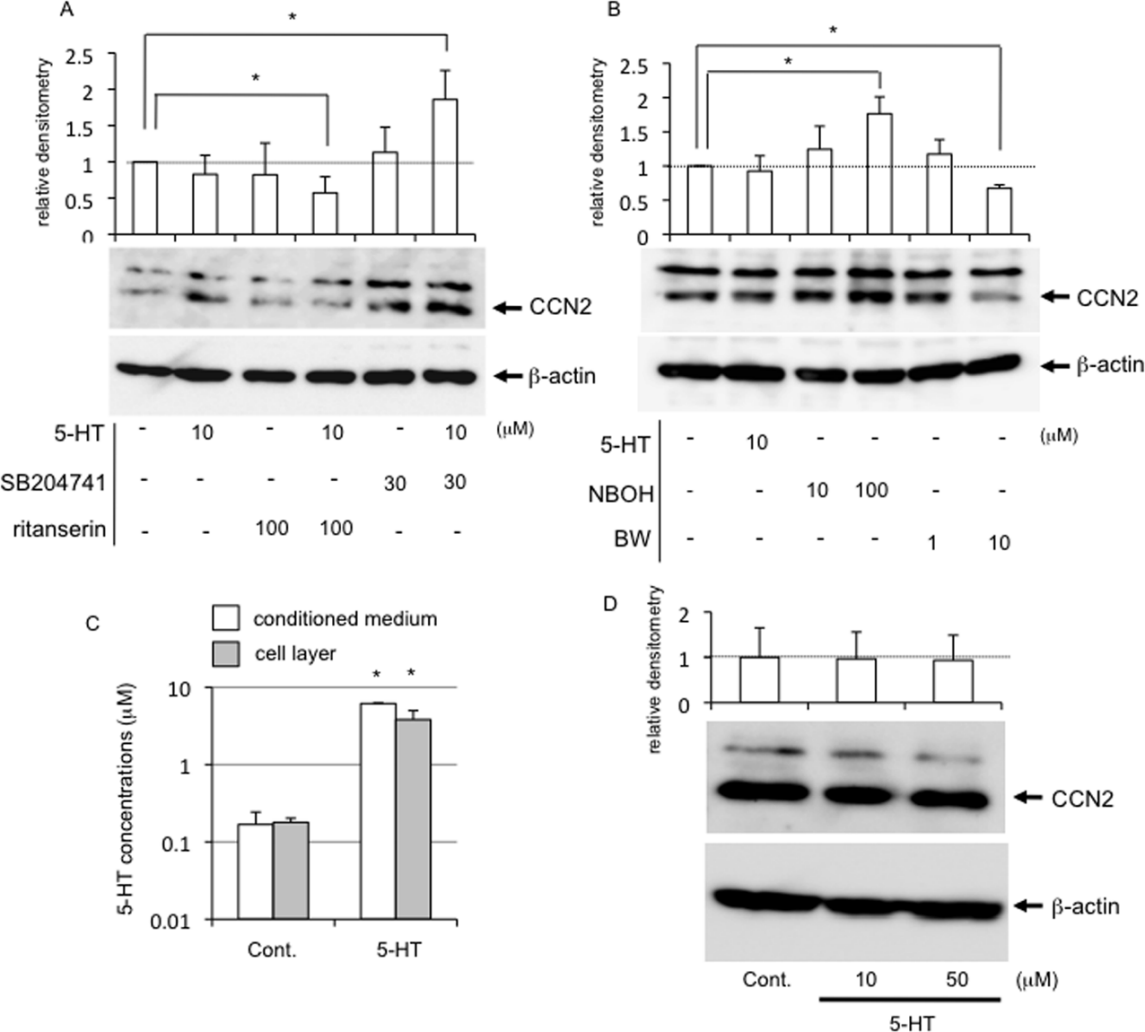
Protein production of CCN2 regulated by 5-HT signaling via 5-HT_2A_R and 5-HT_2B_R. HCS-2/8 cells were grown until they had reached confluence. (A) The cells were treated with vehicle control (PBS and/or DMSO), 5-HT, ritanserin alone, the combination of 5-HT and ritanserin, SB204741 alone, or the combination of 5-HT and SB204741 at the indicated dose. Cell lysates were prepared 24 h later, and Western blot analysis was performed with anti-CCN2 and β-actin antibodies. The graph indicates relative densitometry to untreated controls (ratio = 1.0; dotted line) from 5 independent cultures and analyzed by Bonferroni’s test, and *p* < 0.05 (*) was considered significant. (B) HCS-2/8 cells were treated with 5-HT alone (10 μM), NBOH-2C-CN (10 μM or 100 μM) or BW723C86 (1 μM or 10 μM) for 24 h. Then cell lysates were prepared, and Western blot analysis was performed. Relative densitometry (untreated control = 1.0; dotted line) from 5 independent cultures are presented and analyzed by Bonferroni’s test, and *p* < 0.05 (*) was considered significant. (C) HCS-2/8 cells were treated with 5-HT at the concentration of 10 μM for 24 h, and the cell culture supernatant and cell layer fraction were harvested. Quantification of 5-HT was performed by using an ELISA system. The concentration of 5-HT produced by HCS-2/8 cells was determined by subtracting the 5-HT concentration in fresh media from that in conditioned media. Results are presented as the mean and standard deviations of 3 independent cultures and analyzed by Bonferroni’s test, and *p* < 0.05 (*) was considered significant. (D) HCS-2/8 cells were treated with 5-HT at the concentration of 10 and 50 μM for 24 h, and Western blot analysis was performed. The graph indicates relative densitometry to untreated controls (ratio = 1.0; dotted line) from 3 measurements and analyzed by Dunnett’s test, and there was no significant difference.

Since we already confirmed that both 5-HT_2A_R and 5-HT_2B_R were expressed in HCS-2/8 cells, we next investigated the effect of 5-HT on the chondrocyte differentiation in HCS-2/8 cells. To clarify the effect of 5-HT via each receptor, we used each agonist or antagonist of 5-HT_2A_R and 5-HT_2B_R. Firstly, we examined the effect of 5-HT_2B_R signaling on chondrocyte differentiation. After HCS-2/8 cells had reached confluence, they were treated with BW723C86, which is a 5-HT_2B_R agonist, for 12 h (Fig 2A) and 24 h (Fig 2B). Total RNA was collected, and real-time PCR analysis was performed by using the specific primers for type II collagen (*COL2a1*), aggrecan (*ACAN*) and CCN2 (*CCN2)*, which are markers of chondrocyte differentiation. As a result, the gene expression of *CCN2* was dramatically decreased by BW723C86 (Fig 2A; c), whereas the gene expression of *COL2a1* and *ACAN* showed a tendency to decrease and increase, respectively (Fig 2A; a and b). However, as shown in Fig 2B, the gene expression of all of the genes examined showed the tendency toward a decrease by BW723C86 treatment for 24 h. Especially, *CCN2* expression was significantly decreased (Fig 2B; c). These data suggest that 5-HT signaling regulates the gene expression of CCN2 in chondrocytes. To confirm this hypothesis, we next used ritanserin and SB204741, which are 5-HT_2A_R and 5-HT_2B_R antagonists, respectively. When HCS-2/8 cells were treated with ritanserin, SB204741, the combination of 5-HT with ritanserin or SB204741 for 12 h (Fig 3A) and 24 h (Fig 3B), the gene expression of *COL2A1*, *ACAN*, and *CCN2* was investigated. As shown in Fig 3A, the expression of none of these genes was significantly affected by treatment with 5-HT alone; and treatment with ritanserin alone, SB204741 alone, or the combination of 5-HT and ritanserin or SB204741 also had no effect on *COL2A1* and *ACAN* expressions as compared with the treatment with vehicle. However, only the *CCN2* gene expression was significantly increased by treatment with SB204741 alone, or the combination of 5-HT and SB204741 as compared with that by the treatment with vehicle (Fig 3A; c). Furthermore, we examined the effects of these antagonists and the combination with 5-HT after 24 h-treatment (Fig 3B). Although *COL2A1*, *ACAN* and *CCN2* gene expression was not affected by treatment with 5-HT alone for 24 h, all gene expression was decreased by treatment with ritanserin alone or the combination of 5-HT and ritanserin. On the other hand, treatment with SB204741 alone or the combination of 5-HT and SB204741 increased *ACAN* and *CCN2*, but not *COL2a1* expressions, compared with the treatment with vehicle (Fig 3B). Since it is well known that both *COL2a1* and *ACAN* are under the positive regulation by CCN2 [10, 16, 25], these results suggest that 5-HT affects chondrocyte differentiation via the regulation of CCN2. To confirm these results at the protein levels, we performed Western blot analysis of HCS-2/8 cells treated with ritanserin, SB204741, and the combination with 5-HT and these antagonists. As shown in Fig 4A, Western blotting revealed that CCN2 production was inhibited by the combination of 5-HT and ritanserin and was promoted by the combination of 5-HT and SB204741, compared with the treatment with vehicle. Furthermore, to confirm these results in a more direct way, after treatment with the NBOH-2C-CN and BW723C86, which are 5-HT_2A_R and 5-HT_2B_R agonists, respectively, we evaluated the CCN2 production in HCS-2/8 cells. As shown in Fig 4B, NBOH-2C-CN increased CCN2 production; and BW723C86 contrarily decreased it. These findings indicate that signalings via 5-HT_2A_R and 5-HT_2B_R are counteracting each other in CCN2 production. Therefore, next, to test if the outcome of the counteraction depends on extracellular or intracellular 5-HT level, we quantified the amount of 5-HT in the conditioned media and cell layer of HCS-2/8 cells treated with or without 5-HT by ELISA. As shown in Fig 4C, the concentration of 5-HT secreted by HCS-2/8 cells was approximately 0.2 μM, and it came up to approximately 6.0 μM upon the treatment with 5-HT. On the other hand, intracellular 5-HT level (cell layer) in the absence and presence of 5-HT was 0.1 μM and 4.0 μM, respectively (Fig 4C). These results indicate that extracellular and intracellular 5-HT level is increased by the addition of 5-HT, suggesting that HCS-2/8 cells have the ability of synthesis and reuptake of 5-HT. However, both gene expression and protein production of CCN2 was not affected by treatment with 5-HT alone (Figs 3A, 3B, 4A and 4B). Taken together, these findings suggest that CCN2 production by 5-HT is independent of extracellular and intracellular 5-HT level. In fact, we examined CCN2 production in HCS-2/8 cells treated with 5-HT at concentration of 10 and 50 μM, respectively. As shown in Fig 4D, either 5-HT at 10 or 50 μM had no effect on CCN2 production. In addition, fluvoxamine, which is an inhibitor of 5-HT reuptake, also did not affect CCN2 production (data not shown). These results support this hypothesis that the counteraction in CCN2 production through 5-HT_2A_R and 5-HT_2B_R is independent of extracellular and intracellular 5-HT level.

### Calcium influx was induced by treatment with 5-HT and each agonist and activation of Akt and PKCε were important for differential signaling through 5-HT_2A_R and 5-HT_2B_R

**Fig 5:**
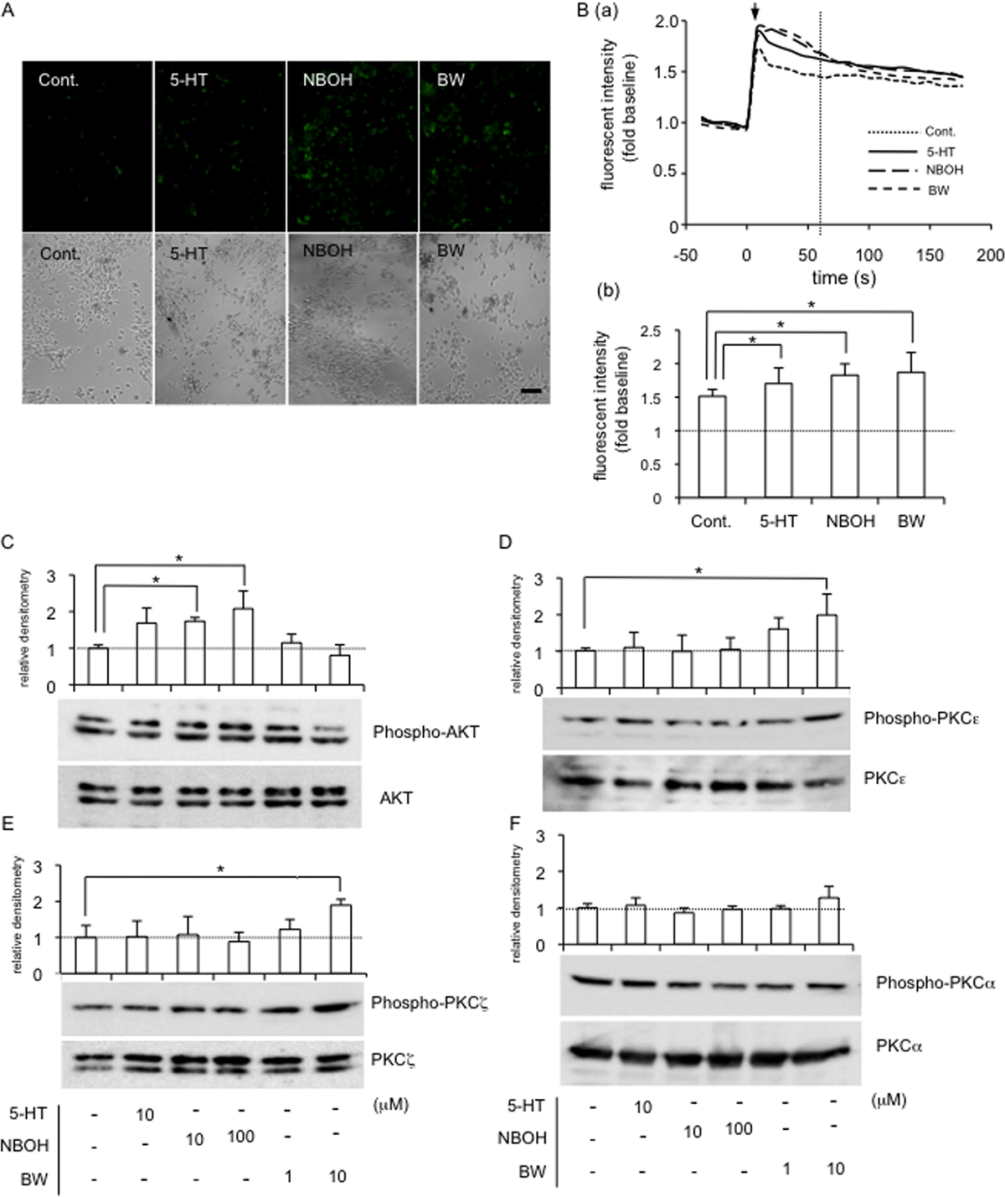
Activation of Ca^2+^ influx, and phosphorylation of Akt and PKCs in HCS-2/8 cells stimulated with 5-HT, and agonists of 5-HT_2A_R and 5-HT_2B_R. (A) After HCS-2/8 cells had reached sub-confluence, the cells were pre-treated with Fluo-4AM (final concentration; 3 mmol/l) in a recording medium at 37°C. After 20 min, the culture medium was replaced with recording medium without Fluo-4AM; and these cells were then treated with 5-HT (10 μM), NBOH-2C-CN (100 μM) or BW723C86 (10 μM) for 1 min. Photographs of the cells were taken under a fluorescence microscope (upper panels). The same field was visualized by phase-contrast microscopy (lower panels). The bar represents 50 μm. (B; a) Time course of fluorescence intensity measured by using a Fluo-4 AM in HCS-2/8 cells treated with 5-HT, NBOH-2C-CN or BW723C86. The ordinate indicates the ratio of fluorescence intensity with respect to untreated sample (ratio = 1.0). Arrow indicates stimulation point (time = 0). (b) Bar graph shows the ratio of fluorescent intensity of each group at 60 seconds after treatment (dotted line in panel “a”). Results are presented as the mean and standard deviations of 8 independent cultures and analyzed by Bonferroni’s test, and p < 0.05 (*) was considered significant. (C-F) After HCS-2/8 cells had reached confluence, the cells were treated with 5-HT or the indicated agonist at the final concentrations shown. After 5 min, cell lysates were prepared; and Western blot analysis was then performed with antibodies recognizing the indicated proteins. (C) The level of phosphorylated Akt (n = 3) was increased by the treatment with NBOH-2C-CN (10 μM and 100 μM) and (D) PKCε (n = 4) and (E) PKCζ (n = 3) phosphorylation levels were increased by the treatment with BW723C86 (10 μM). (F) The level of phosphorylated PKCα (n = 3) showed no change. The total amounts of conventional PKCα (PKCα), novel PKCε (PKCε), and atypical PKCζ (PKCζ) remained unchanged by any treatment. The graph indicates relative densitometry (untreated control = 1.0; dotted line) from 3 measurements and analyzed by Dunnett’s test, and *p* < 0.05 (*) was considered significant.

It is well-known that 5-HT_2_ receptors belong to the G-protein-coupled receptor superfamily, which is characterized by 7 membrane-spanning hydrophobic regions [6, 31]. When these receptors interact with a ligand, the heterotrimeric G proteins (composed of α-, β-, γ-subunits) are activated by exchange of guanosine triphosphate (GTP) for guanosine diphosphate (GDP), and the Gα-GTP complex dissociates from receptor and Gβγ complex [31, 32]. Then, both Gα-GTP and Gβγ activate downstream effectors. Gα-subunit family is divided into 4 groups based on subunit sequence homology, G_s_α, G_i/o_α, G_q/11_α and G_12/13_α [32]. Especially, 5-HT_2_ receptors belong to Gq-coupled receptor family and G_q_α-GTP complex activates phospholipase C (PLC) [31]. The activated PLC produces or modulates diacylglycerol (DAG) and inositol 1,4,5-triphosphate (IP_3_). Then DAG and IP_3_ are attributed to the activation of protein kinase C (PKC) and the release of Ca^2+^ stores in the endoplasmic reticulum, respectively [31]. Moreover, Gβγ subunits can activate phosphoinositide-3 kinase (PI3K), and PI3K induces the phosphorylation of Akt (also termed protein kinase B) [33]. Therefore, we firstly examined whether or not calcium ions were released into the cytosol by stimulation with each agonist. As shown in Fig 5A, more green fluorescent dots, which represent calcium influx, were detected in the cells treated with either 5-HT, NBOH-2C-CN (a 5-HT_2A_R agonist) or BW723C86 (a 5-HT_2B_R agonist) than in those incubated with the vehicle. Next, intracellular Ca^2+^ fluorescence intensity using a Fluo-4 AM was measured in HCS-2/8 cells treated with 5-HT, NBOH-2C-CN or BW723C86. As shown in Fig 5B (a) and (b), fluorescence intensity was increased rapidly by the addition of either 5-HT, NBOH-2C-CN or BW723C86 as well as vehicle, however, the fluorescence intensity was stronger with the treatment with 5-HT and both agonists at 60 seconds after treatment (dotted line) than the control. These findings suggest that Ca^2+^ influx into the HCS-2/8 cells was induced by either the signaling via 5-HT_2A_R or that via 5-HT_2B_R. Since both agonists stimulated Ca^2+^ influx into the cells, we next investigated the phosphorylation of Akt and PKCs, which are downstream kinases potentially activated by these agonists. As shown in Fig 5C, Akt was activated by NBOH-2C-C, while a novel PKC, PKCε, and an atypical PKC, PKCζ, was activated by BW723C86 (Fig 5D and 5E). A conventional PKC, PKCα, showed no changes by the treatment with either agonist (Fig 5F). These findings suggest that Akt and PKCs play important roles in determining which intracellular signaling pathways are activated by NBOH-2C-CN and BW723C86, respectively.

### Multiple MAPKs were phosphorylated in HCS-2/8 cells treated with either agonist, and BW723C86-regulated CCN2 production was mediated by ERK1/2 and JNK

**Fig 6:**
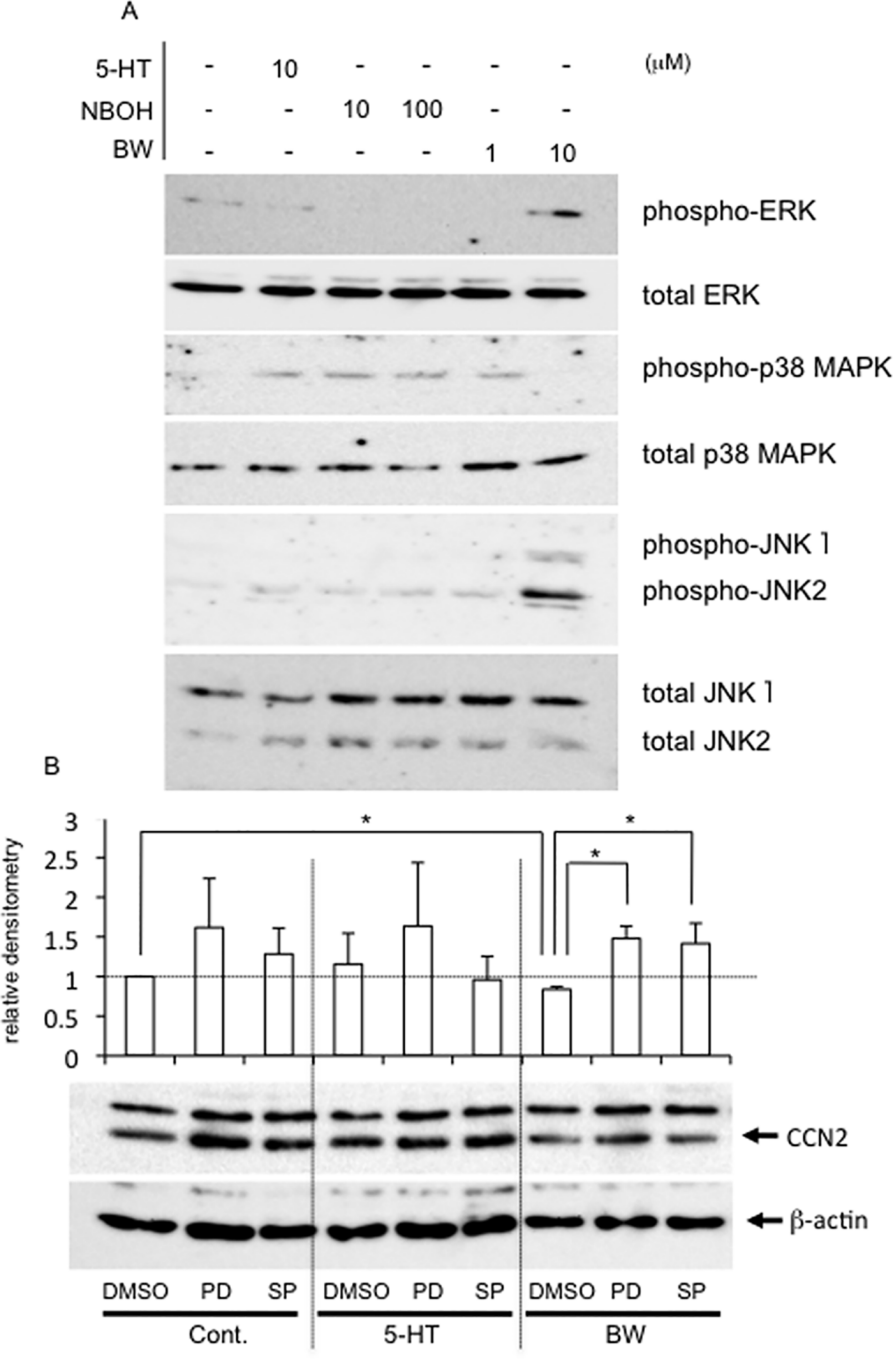
Activation of MAPKs in HCS-2/8 cells treated with 5-HT or agonist of each 5-HT_2_ receptor and rescue effect of inhibitors of ERK1/2 and JNK on the BW723C86-decreased CCN2 production. (A) HCS-2/8 cells were grown until they had reached confluence. Then, the cells were treated with 5-HT, NBOH-2C-CN or BW723C86 at the indicated concentrations. After 15 min, cell lysates were prepared; and Western blot analysis was performed with the antibodies against the indicated proteins. The levels of phosphorylated ERK1/2 and JNK were increased by the treatment with BW723C86 at a concentration of 10 μM. In contrast, the levels of phosphorylated p38 MAPK were increased by the treatment with NBOH-2C-CN at the concentrations of 10 μM and 100 μM. (B) HCS-2/8 cells were grown until they had reached confluence. Then, when the cells were treated with 5-HT (10 μM) or BW723C86 (10 μM), PD98059 (MEK1 inhibitor; 50 μM) or SP600125 (JNK inhibitor; 50 μM) was applied to the cultures simultaneously. After 24 h, the cell lysates were prepared; and Western blot analysis was performed with anti-human CCN2 rabbit serum and β-actin antibody. When HCS-2/8 cells were treated with 5-HT, PD98059 or SP600125 had no effects. In contrast, when the cells were treated with BW723C86, CCN2 production was rescued by either PD98059 or SP600125. The graphs give the results of Western blotting using anti-CCN2 antibody and quantified by densitometric analysis, with normalization by the levels of β-actin. The ordinate indicates the fold change relative to untreated controls (ratio = 1.0; dotted line). The graph indicates relative densitometry (untreated control = 1.0; dotted line) from 6 independent cultures and analyzed by Bonferroni's test, and *p* < 0.05 (*) was considered significant.

Next, we investigated how MAPKs, which are downstream targets of Akt and PKC signaling, were affected by each agonist. As shown in Fig 6A, treatment with BW723C86 enhanced the phosphorylation of both ERK1/2 and JNK presumably resulting in decreased CCN2 production. Also, treatment with NBOH-2C-CN enhanced phosphorylation of p38 MAPK as well as JNK, presumably as a result, increasing CCN2 production (Fig 6A). To determine if MAPKs were involved, we examined whether or not this BW723C86-decreased CCN2 production could be prevented by pretreatment with inhibitors of either ERK1/2 or JNK. We observed earlier that PD98059 (ERK1/2) and SP600125 (JNK) inhibit the phosphorylation of ERK1/2, and JNK, respectively, in HCS-2/8 cells under the same conditions [34]. As shown in Fig 6B, CCN2 production in controls and in 5-HT treated cells was not affected by PD98059 or SP600125, whereas CCN2 production in the presence of BW723C86 was increased by the treatment with ERK inhibitor PD98059 and JNK inhibitor SP600125. These results support our contention that BW723C86 decreases CCN2 production through the activation of ERK1/2 and JNK.

### Immunolocalization of 5-HT_2A_R and 5-HT_2B_R in the growth plate and articular cartilage tissues

**Fig 7:**
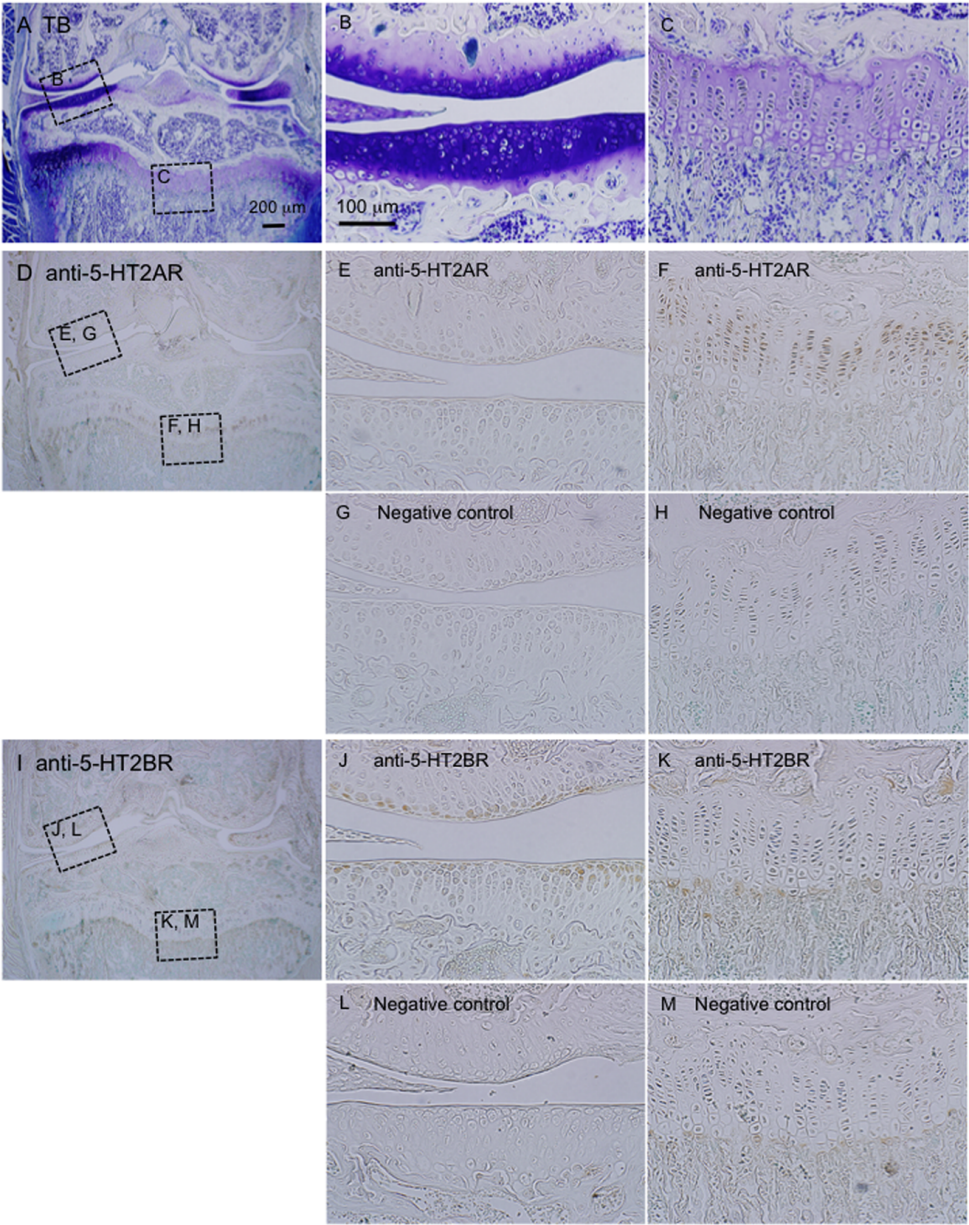
Immunohistochemical analysis of whole knee joints from 60 day-old male mice by use of anti-5-HT_2A_R and 5-HT_2B_R antibodies. (A-C) Sections of the frontal knee joints were stained with toluidine blue, and cartilage tissues showed metachromatic staining. The areas surrounded by the boxes are enlarged in “B” (articular cartilage tissues) and “C” (growth plate). (D-H) In the low-power magnification view of the knee joint stained with anti-5-HT_2A_R (D) the areas surrounded by the boxes are enlarged (E, H). Images of “E” and “F” represent articular cartilage tissues (E) and the growth plate (F). A serial section was stained with a non-immune antibody as a negative control, and images of the same areas as seen in “E” and “F” are shown in “G” and “M”, respectively. The immunoreactivity for 5-HT_2A_R was detected in cells from the proliferating to prehypertrophic regions of the growth plate. (I-M) The knee joint stained with anti-5-HT_2B_R. In the low-power-magnification view (I), the areas indicated by the boxes are enlarged in “J” and “K”. Images in “J” and “K” represent articular cartilage tissues (J) and the growth plate (K). Images in “L” and “M” represent the same areas as seen in “J” and “K,” respectively, in a serial section stained with a non-immune antibody as a negative control. The immunoreactivity for 5-HT_2B_R was detected in the surface layer of articular cartilage tissues. The sizes of scale bars are indicated.

Because our results indicate that CCN2 production is regulated by 5-HT signaling via 5-HT_2A_R and 5-HT_2B_R in HCS-2/8 cells, we next investigated whether or not 5-HT_2A_R and 5-HT_2B_R were localized in cartilage tissues, including articular cartilage and growth plate. Little immunoreactivity for 5-HT_2A_R was detected in the articular cartilage tissue (Fig 7E), but it was strongly detected in chondrocytes from the proliferating to prehypertrophic regions of the growth plate (Fig 7F). On the other hand, 5-HT_2B_R was strongly localized in the surface of the articular cartilage (Fig 7J), whereas it was not detected in the growth plate (Fig 7K). Previously we reported that CCN2 expression is strongly detected in the prehypertrophic zone of growth plate [14] and slightly detected in articular cartilage tissue [15]. Therefore, taken together with these data and the results shown in Figs 2 and 3, these findings suggest that the distribution of 5-HT_2A_R and 5-HT_2B_R is highly consistent with the gene expression pattern of CCN2 in the growth plate and articular cartilage.

## Discussion

In this study, we focused on the effect of 5-HT on CCN2 production in cartilage tissue, which had not been previously recognized as a target tissue of 5-HT. We first examined the gene expression of the 5-HT_2A_R, 5-HT_2B_R, and 5-HT_2C_R in human chondrocytic cell line HCS-2/8 and demonstrated that 5-HT_2A_R and 5-HT_2B_R genes were expressed, but 5-HT_2C_R gene was not (Fig 1A). In addition, we confirmed the gene expression of *TPH-1* in HCS-2/8 cells (Fig 1A). Furthermore, we showed that HCS-2/8 cells produced 5-HT and took it up into the cells (Fig 4C). These findings imply that chondrocytes might be capable of synthesis and degradation of 5-HT. It is well-known that 5-HT is produced by neurons in the CNS [1–3] and that most of the 5-HT present in peripheral tissues is produced by cells in the gut [1–3]. Gut-derived 5-HT is taken up by platelets, and the 5-HT thus enters the circulation [1–3]. When platelets are activated and exhibit strong vasoconstrictive properties, 5-HT is released and is involved in various biological events [1–3]. However, since cartilage tissues have no nervous and vascular systems, we consider that CCN2 production is directly regulated by local 5-HT, which is produced by chondrocytes and acts in an autocrine and paracrine manner. Therefore, to investigate the role of 5-HT_2_ receptors, which expressed in cartilage anlage [7], we applied ritanserin and SB204741, which are antagonists of 5-HT_2A_R and 5-HT_2B_R, respectively, to the culture of HCS-2/8 cells. At 12 h post-treatment, only CCN2 gene expression was significantly increased in HCS-2/8 cells treated with SB204741 alone or the combination of 5-HT and SB204741, and showed a tendency to decrease by the treatment with ritanserin alone, or the combination of 5-HT and ritanserin (Fig 3A). These findings suggest that 5-HT signaling via 5-HT_2_ receptors regulates CCN2 expression. Serum-derived 5-HT in culture medium may have supported the effects of antagonist alone. To confirm the effect of 5-HT signaling via 5-HT_2_ receptors on the regulation of CCN2, NBOH-2C-CN and BW723C86, which are agonists of 5-HT_2A_R and 5-HT_2B_R, respectively, were applied to the cells. Recently, NBOH-2C-CN was reported to be a novel 5-HT_2A_R agonist with a *p*Ki of 1.3 nM for 5-HT_2A_R and 100-fold more selective for it than for 5-HT_2C_R [22]. However, this compound at low concentrations of 10−8–10^−7^ M showed no effect in HCS-2/8 cells (data not shown). It was previously indicated that 5-HT_2_ receptor ligands can have different affinities for the same receptors in different tissues [35], so it is possible that the *p*Ki values for this compound may differ in cartilage tissues from the reported one. Therefore, we tested NBOH-2C-CN at higher concentrations. As was shown in Fig 4B, when NBOH-2C-CN was applied to cultures of HCS-2/8 cells, CCN2 production was increased; and treatment with BW723C86, which is a 5-HT_2B_R agonist, decreased CCN2 production. These findings suggest that 5-HT up- and down-regulates CCN2 via 5-HT_2A_R and 5-HT_2B_R, respectively. Therefore, transduction of 5-HT signaling into the cells through the simultaneous activation of both 5-HT_2A_R and 5-HT_2B_R could occasionally result in apparently no effect on CCN2 expression or production. It should be also noted that 5-HT_2A_R and 5-HT_2B_R may have specific ligands other than 5-HT, since increased extracellular 5-HT level resulted in no increase in CCN2 production.

Next, we investigated the intracellular signaling pathways of 5-HT in HCS-2/8 cells. It is known that 5-HT receptors are G-protein-coupled receptors, which generally mediate calcium signaling [6]. Therefore, we suspected that Ca^2+^ flowed into the cytoplasm of HCS-2/8 cells upon 5-HT, NBOH-2C-CN, or BW723C86 treatment. Indeed, Ca^2+^ influx was detected by treatment with 5-HT and either agonist (Fig 5A and 5B). As Ca^2+^ influx into the cells was induced by both 5-HT and agonists, we next analyzed the downstream mediator Akt and PKC, which are involved in the 5-HT signaling in chondrocytes. Akt is known as protein kinase B and is phosphorylated by phosphatidylinositol 3, 4, 5-triphospate (PIP3), which is produced by PI3K [31]. In this study, the level of phospho-Akt was increased by treatment with the agonist of 5-HT_2A_R, and that of phospho-p38 MAPK was also increased (Figs 5 and 6), which resulted in increased CCN2 production. Previously, we reported that PI3K-Akt pathway is involved in the hypertrophic differentiation of chondrocytes [34], and other researchers reported that the phosphorylation of p38 MAPK signaling is required for hypertrophic differentiation of chondrocytes [36]. Consistent with these reports, our present results indicated that 5-HT_2A_R was distributed in cells from the proliferating to pre-hypertrophic zones in the growth plate (Fig 7). Together with the results of our previous study showing that CCN2 is highly expressed in pre-hypertrophic chondrocytes *in vivo*, it is quite reasonable that 5-HT-5-HT_2A_R signaling induces CCN2 production in pre-hypertrophic zone of growth plate to promote hypertrophic differentiation.

PKCs are composed of several isoforms that are divided into 3 basic classes, i.e., conventional, novel, and atypical, according to the structure of their regulatory domains [37]. The conventional PKC (cPKC) includes PKCα; and the novel and atypical PKCs include PKCε and PKCζ, respectively [37]. Previously, it was reported that chick limb bud mesenchymal cells express PKCα, PKCε and PKCζ isoforms during chondrocyte differentiation [38], suggesting that PKCs have important roles in chondrocyte differentiation. In this study, when the agonist of 5-HT_2B_R was added to HCS-2/8 cells, phospho-PKCε and phospho-PKCζ were increased, but phospho-PKCα was not affected (Fig 5D–5F), and ERK1/2, which is a downstream kinase of PKCs, was also phosphorylated (Fig 6). As a result, CCN2 production was oppositely decreased (Fig 4). Since it was reported earlier that ERK1/2 and p38 MAPK conversely regulate chondrogenesis by modulating the gene expression of adhesion molecules [39], the 5-HT-5-HT_2B_R-ERK1/2 pathway and 5-HT-5-HT_2A_R-p38 MAPK pathway may well differentially regulate chondrocyte differentiation by modulating CCN2 production. In a previous study, we revealed that CCN2 production in articular cartilage tissues is less than that in the growth plate [14, 15]. In this context, we showed that 5-HT_2B_R was localized in the surface layer of articular cartilage in this study (Fig 7). Collectively, these data suggest that CCN2 production may be down-regulated by 5-HT via 5-HT_2B_R, which is localized in the surface layer of articular cartilage. Furthermore, we also confirmed that JNK in the HCS-2/8 cells was phosphorylated by the treatment with the 5-HT_2B_R agonist (Fig 6). Since others also reported that phosphorylation of JNK is involved in the promotion of CCN2 production in fibroblasts [40], further study is needed to identify the upstream kinase that transmits 5-HT-5-HT_2B_R signaling to JNK.

**Fig 8:**
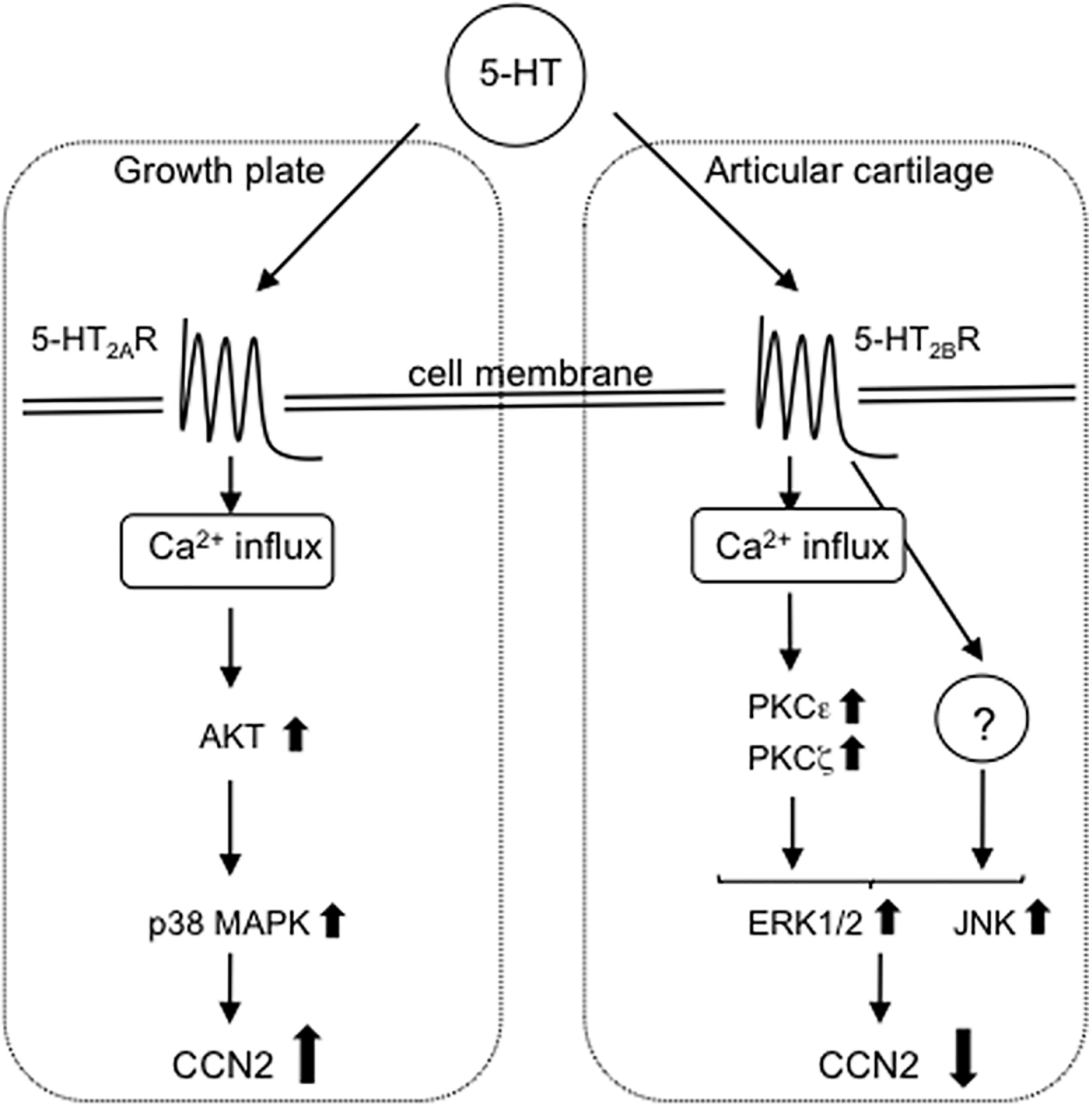
Schematic representation of possible intracellular 5-HT signaling pathways in chondrocytes. Results newly obtained in the present study are summarized. Since 5-HT_2A_R is localized in the growth plate, 5-HT signaling via 5-HT_2A_R induces Ca^2+^ influx. Then, p38 MAPK is activated by phosphorylated Akt; as a result, CCN2 production is increased. On the other hand, since 5-HT_2B_R is localized in articular cartilage tissues, 5-HT signaling via 5-HT_2B_R induces Ca^2+^ influx similar to 5-HT signaling via 5-HT_2A_R. Then, the phosphorylated ERK1/2 level is increased through both activation of PKCε and PKCζ; as a result, CCN2 production is decreased. Furthermore, independent of PKC, 5-HT may transmit a certain unknown signal to JNK, which also inhibits the CCN2 production in chondrocytes.

In summary, we demonstrated herein that 5-HT played a direct regulatory role in CCN2 production as a novel 5-HT function in chondrocytes. As schematized in Fig 8, 5-HT signaling via 5-HT_2A_R promotes Akt phosphorylation followed by the activation of p38 MAPK, resulting in increased CCN2 production. On the other hand, the signaling through the activation of 5-HT_2B_R induces the phosphorylation of both PKCε and PKCζ, which increases phospho-ERK1/2 and phospho-JNK levels, resulting in decreased CCN2 production. Based on these findings and our immunohistochemical data indicated in Fig 7, we propose that 5-HT has distinct roles in CCN2 production between the growth plate and articular cartilage tissues. However, because the results obtained from in the present study using HCS-2/8 cells may not completely represent corresponding events in chondrocytes from growth plate or articular cartilage, further study with primary cells is needed for the future.

## Supporting information

S1 Checklist NC3Rs ARRIVE guideline checklist 2014. (PDF)

## Abbreviations

5-HT: 5-hydroxytryptamine
TpH: tryptophan hydroxylase
5-HTpD: 5-hydroxytryptophan decarboxylase
HCS-2/8: human chondrocytic cell line-2/8
5-HT_2_Rs: 5-HT_2_ receptors
5-HTT: 5-HT transporter
CNS: central nervous system
EC cells: enterochromaffin cells
CCN2/CTGF: CCN family protein 2/connective tissue growth factor
BW: BW723C86
NBOH: NBOH-2C-CN
SB: SB206553
ERK1/2: extracellular signaling regulated protein kinase1/2
JNK: c-Jun N-terminal kinase
PKC: protein kinase C
